# EXAMINING SOCIODEMOGRAPHIC DATA REPORTING REQUIREMENTS IN STATE DISEASE SURVEILLANCE SYSTEMS

**Published:** 2021

**Authors:** SAMANTHA BENT WEBER, AMANDA MORELAND, RACHEL HULKOWER, TARA RAMANATHAN HOLIDAY

**Affiliations:** * Public Health Analyst at the Office of Public Health Law Services, Center for State, Tribal, Local, and Territorial Support, CDC.; ** Public Health Analyst with DRT Strategies, supporting the Office of Public Health Law Services, Center for State, Tribal, Local, and Territorial Support (CSTLTS), CDC.; *** Public Health Advisor at the National Center for Injury Prevention and Control, CDC.; **** Public Health Analyst at the Office of Public Health Law Services, Center for State, Tribal, Local, and Territorial Support, CDC.

## Abstract

Law plays an important role in the collection of data related to disease and injury in a population. A robust system of laws sets out requirements for the collection, analysis, and dissemination of disease reporting data from local, state, territorial, and federal public health institutions. Occurrence of disease, including outbreaks of novel infectious agents like coronaviruses, influenza viruses, and others that have arisen in recent years, often require epidemiologists and others to understand not only the etiology and specific context of diseases and conditions, but also the trajectory of their spread among and across communities. Capturing sociodemographic data is critical to identifying the disproportionate impacts of diseases and conditions on diverse populations and determining an appropriate public health response. This Article discusses a legal epidemiological scan of state disease reporting laws that require the capture and reporting of sociodemographic information. Analyzing these state laws can serve as a potential starting point to assist in understanding why gaps in data exist and can help address these challenges in anticipation of future disease outbreaks or spread.

## I. Background on Disease Reporting and Surveillance

Since the inception of disease surveillance in the United States, the legal foundation for tracking and monitoring the occurrence of disease and injury within a population has been established through a combination of laws and policies at various governmental levels. The Centers for Disease Control and Prevention (CDC) houses the country’s most expansive disease surveillance systems, including the National Notifiable Diseases Surveillance System (NNDSS). The NNDSS receives case-level data from fifty-seven public health jurisdictions, including states, localities, and territories, for the approximately 120 diseases and conditions that comprise the National Notifiable Conditions.^1^ This disease surveillance system relies upon the jurisdictions’ voluntary submission of data on infectious and noninfectious diseases and conditions that have been deemed *notifiable*.^2^ Jurisdictions remove information that would identify the individual before submitting these data to CDC for use by the disease-specific programs, which support state, local, and territorial public health departments.^3^ This surveillance system plays a critical role in monitoring, preventing, and controlling the incidence of disease and injury across the country.

Foundational to the NNDSS are the state, local, and territorial laws and policies that require healthcare providers, healthcare facilities, laboratories, and others to submit information on *reportable* diseases and conditions to the state, local, or territorial public health department.^4^ Each state, locality, and territory has the legal authority to enact laws that determine which conditions are reportable within its own jurisdiction, in addition to who must report and the methods for reporting.^5^ As such, the list of mandatory reportable diseases and the manner in which they are reported vary by jurisdiction.^6^ Many, though not all, reportable diseases are also on the NNDSS list of Nationally Notifiable Conditions.^7^ The collection and dissemination of disease reporting data from each jurisdiction is essential to the compilation of case notification information that comprises NNDSS and other national surveillance systems.

## II. The Importance of Sociodemographic Data in Disease Surveillance

Disease surveillance information is critical to designing and implementing strategies to protect the public’s health.^8^ Public health surveillance through NNDSS and other systems allows epidemiologists to detect, track, and respond to indicators of disease.^9^ For example, some outbreaks involve new or reemerging infectious diseases, which may make them more difficult to predict or control.^10^ Measuring and monitoring trends and changes to diseases, including their incidence and the effectiveness of disease control measures, requires the availability of information beyond just the disease name and clinical data.^11^

The collection of sociodemographic data is essential to disease control efforts. Demographic data, or characteristics of individuals within a population, such as age, race, sex, pregnancy status, employment status, or occupation, can help to identify traits among those who are most affected by a disease, as well as how the disease spreads across populations.^12^ This Article uses the parallel term “sociodemographic” to refer to these data as it implicates a larger set of social, societal, and structural factors that frequently correspond with population characteristics and contribute to health outcomes.^13^ These factors are characterized in public health practice as social determinants of health.^14^ Sociodemographic data help identify populations that experience disproportionate impacts of disease and may help provide indicators of the social, environmental, economic, and other conditions in which those populations live.^15^ These data are also critical for examining the environments that contribute to disparate health outcomes across populations.^16^ Equally important is identifying populations that not only may experience excess morbidity and mortality, but also may confront fear, stigma, and discrimination in their daily lives and as a result of their health status.^17^

Disease reporting laws help to facilitate the collection of data that can be used to explore the impact of disease on populations that experience health-harming societal disadvantages on account of sociodemographic factors like race, ethnicity, socioeconomic status, occupation or industry, pregnancy status, and geographic location, among others.^18^ Moreover, as public health practice continues to establish frameworks to advance equity in health outcomes, disease surveillance can play an important role in building an evidence base about the association between societal conditions and greater morbidity and mortality in certain communities.^19^ Indeed, as “[p]ublic health work is built on disease or injury surveillance data … the elements collected in data systems are what shape that foundation.”^20^

## III. Laws That Address Sociodemographic Data Reporting

Sociodemographic data have long been recognized as important dimensions of disease reporting, but the inadequate collection of these data has posed a persistent, yet underexamined, challenge.^21^ For example, during the period between 2006 and 2010, about thirty percent of cases reported to NNDSS lacked information about race and about fifty-one percent of reported cases lacked information about ethnicity.^22^ In another example, a search of the literature between 2006 and 2015 indicated that occupation and workplace data were available for a few specific industries, rather than across labor forces and workplaces; accordingly, limited research has thus far examined the conditions that may place workers at increased risk of exposure to infectious diseases.^23^

These examples of gaps in data collection raise questions about the extent to which state disease reporting laws require the reporting of sociodemographic data and whether such a mandate might help to mitigate reporting gaps. While the lack of available sociodemographic data cannot be attributed to a single factor, one reason that jurisdictions do not routinely collect this information might lie with the lack of legal incentive to do so. As described above, the state-level mandatory reporting of identified diseases and conditions facilitates the collection of data necessary to assess the burden of disease and injury within and across populations.^24^ However, gaps in these data may also be due, in part, to the substantial variations in reporting requirements across jurisdictions.^25^ These variations are significant for two main reasons: first, they can influence the manner in which a public health response unfolds in the face of a widespread outbreak or other public health emergency;^26^ second, they may contribute to gaps in the reporting of data necessary to assess impacts of disease on specific populations.^27^ While law cannot accurately be framed as the sole solution to complex issues related to healthcare capacity and data sharing, law can serve as a tool for understanding the breadth and scope of reporting requirements that health departments follow and implement in carrying out disease reporting.^28^

As a starting point to understanding gaps in the collection of sociodemographic data, researchers at CDC’s Public Health Law Program (PHLP) conducted a legal epidemiological scan of state disease reporting laws in all fifty states, focusing specifically on requirements to report sociodemographic information.^29^ Each state’s legal code includes a chapter—typically located in its health or public health section—consisting of provisions that set out requirements for the reporting of diseases and conditions.^30^ Researchers analyzed these statutes and regulations for the presence or absence of express requirements to submit sociodemographic information.^31^ This study did not assess agency-level policies on disease reporting, which are typically not included in state statutes or regulations. Nor did this study examine the information that health departments request through forms or internet-based portals that might capture sociodemographic data. These types of policies also play a role in sociodemographic data collection and may benefit from future research.^32^

## IV. Findings

Documented gaps in sociodemographic data collection raised questions about whether states have laws imposing requirements for the reporting of sociodemographic data within their disease reporting chapters.^33^ For example, an assessment of disease surveillance laws and their relationship to case reporting of 2009 H1N1 infections found that although forty-nine jurisdictions around the country described encountering no problems related to a lack of legal authority to collect case data and report it to CDC, thirty jurisdictions cited the reason for not collecting sociodemographic data like race, ethnicity, or obesity status as “lack of staff resources or unavailability of data from medical providers.”^34^ This important and broad finding still left an open question about the scope of reporting requirements imposed by state laws.

PHLP’s legal epidemiological scan showed that forty-eight states had statutory or regulatory provisions in their disease reporting chapters that expressly required the reporting of sociodemographic data for at least one reportable disease or condition.^35^ State laws varied in the situations that trigger reporting of sociodemographic data, with jurisdictions either requiring this reporting for *any* finding of a reportable disease or condition or requiring it only for the diagnosis of a specific reportable disease or condition, such as HIV or tuberculosis.^36^ Typically, public health departments collect disease reporting data from those who directly ascertain findings of reportable diseases or conditions, namely healthcare providers, healthcare facilities, and laboratories. Indeed, forty-eight states required healthcare providers or facilities and forty-five states required laboratories to submit sociodemographic data for findings of reportable diseases or conditions.^37^ At the same time, some states also imposed reporting requirements on additional actors such as school administrators,^38^ day care centers,^39^ correctional facilities,^40^ “person[s] in charge of a licensed house of prostitution,”^41^ or “any other person[s] having knowledge of any disease which may threaten the public health.”^42^

State laws also varied in the scope of sociodemographic information required. Forty-seven states listed specific data to be reported, and researchers identified only one state whose laws required sociodemographic reporting for at least one reportable disease without specifying *any* data element.^43^ Forty-six states explicitly required the collecting and reporting of patient names and addresses,^44^ but they varied in their requirements to collect information about race or ethnicity, sex, gender, occupation, or pregnancy status.

PHLP researchers observed that of the forty-seven states with statutes or regulations that required the reporting of specific elements of sociodemographic data, thirty-nine required reporting of a patient’s race or ethnicity.^45^ Eight of the forty-seven states imposed no specific mandate within disease reporting laws to report racial or ethnic information.^46^ Reporting requirements related to sex and gender also varied across these forty-seven jurisdictions. Thirty-five states expressly required the reporting of sex, sixteen required the reporting of gender, and eight required both sex and gender to be reported.^47^ California, for example, separately required the reporting of gender identity and sex assigned at birth.^48^ Forty-six of these forty-seven states specified the reporting of age or date of birth.^49^

Thirteen states required the reporting of pregnancy status for at least one disease or condition.^50^ Conversely, thirty-four of the forty-seven states that specified at least one sociodemographic marker for reporting did not require the reporting of pregnancy status.^51^ Seventeen of the forty-seven states required the reporting of occupation, place of work, or industry when providing patient data; correspondingly, thirty states did not.^52^ Uniquely, New Hampshire required reporting of the patient’s occupation and *place* of occupation.^53^

No states required reporting of citizenship status in their disease reporting laws, and three states required reporting on country of origin.^54^ Only California required the reporting of sexual orientation.^55^

## V. Discussion

As reflected in the findings discussed above, this scan of legal provisions in state disease reporting chapters suggests that states are attuned to the importance of capturing sociodemographic data, particularly with respect to individual patient location, sex, age (and by proxy, date of birth), and race or ethnicity. At the same time, these reporting requirements are not universally mandated across all jurisdictions and across all conditions, which may result in wide variation across the states’ sociodemographic reporting outcomes. These findings, therefore, raise a question about the state of disease surveillance laws: would further promulgation of sociodemographic data reporting requirements help mitigate reporting gaps shown in other studies, and if so, how?

Correspondingly, this scan suggests that there are areas of sociodemographic data reporting that could receive more attention in law. For example, as previously highlighted, thirty-four states did not require the reporting of pregnancy status, and even among states that did, some only required it “if known,” a designation that is prone to ambiguity.^56^ Researchers have emphasized “the unique vulnerabilities of pregnant women and infants to emerging health threats,” as well as the importance of surveillance systems for understanding the impact of disease or emergencies to inform both clinical decision-making and approaches to prevention.^57^ Valuable information for examining and tracking health outcomes among pregnant women can be lost where jurisdictions do not prioritize the collection of these disease reporting data.^58^

As noted previously, a majority of states (thirty of forty-seven) that required the reporting of specific sociodemographic markers did not require the reporting of employment or occupation data, despite the singular risks that workplace settings can pose to disease spread.^59^ Part of this difficulty might arise because diseases associated with the workplace or occupational diseases are typically reported, if at all, via workers’ compensation or other, seemingly disparate, mechanisms.^60^ Some diseases, including influenza and other types of communicable respiratory infections, may not ordinarily be characterized as occupational diseases, but they nevertheless may be connected to specific workplaces and settings.^61^ Accordingly, questions abound about whether state disease reporting systems can be enhanced with a more robust collection of these data, and how law may serve as a tool to help bring this to fruition.

### A. Limitations of the Legal Epidemiological Scan

There are several limitations to these findings. Perhaps most importantly, the law as written on the books does not always capture the policies and practices as they are developed and implemented by public health and health care practitioners. For example, Georgia’s law did not include an express provision in statutes or regulations that required the reporting of sociodemographic data, but its Notifiable Disease/Condition Report Form, which providers and laboratories send to the health department, carves out space for sociodemographic data, such as race, ethnicity, age, sex, and other factors.^62^ The existence of these reporting mechanisms in most states, either through law or public health practice, makes the underreporting of sociodemographic data even more surprising. While the research conducted for the purposes of this Article offers an instructive window into the states’ legislative and executive branch efforts to collect sociodemographic data, it does not provide a comprehensive picture of what departments of public health, or individuals reporting disease, do in practice.

Additionally, while this legal epidemiological scan coded for the presence or absence of an express requirement to report sociodemographic data, a state may only require sociodemographic data to be reported for certain conditions and not for others. For example, Michigan law required practitioners to report a patient’s sex when diagnosing cases of HIV;^63^ accordingly, this study design would characterize Michigan as requiring the reporting of sex, even if it is not required with other diagnoses. Sociodemographic information may also be reported to health department registries, vital records, and other databases—data that can be linked and analyzed to conduct epidemiological analyses—that are outside the scope of disease reporting statutes and regulations reviewed in this scan. Lastly, this study is based on publicly available statutes and regulations as identified by researchers using the WestlawNext legal database. While coding results underwent multiple verification steps for quality assurance, results are subject to the availability of laws and their appropriate inclusion within this dataset. Because this study is intended to serve as a scan of the legal landscape surrounding disease reporting laws, and to shed light on the role of law in creating more comprehensive and informative datasets related to sociodemographic data and burdens of disease, the results of this study should not be taken as legal advice or descriptive of a duty to report.

### B. Considerations for Future Research

The researchers view this scan as a starting point in analyzing the factors contributing to existing gaps in sociodemographic data in the context of disease reporting. Future research might consider several additional factors that are not addressed here. For example, research might utilize a methodology that incorporates a systematic examination of state public health department disease reporting policies, forms, and electronic interfaces used by healthcare providers and laboratories for a more comprehensive picture of disease reporting practices. Such a systematic examination might also expand the universe of potential demographic markers that state laws might seek to capture in disease reporting, such as disability status, potential underlying co-morbidities, or socioeconomic status. It might also stratify demographic markers in relation to specific diseases—in order to precisely capture those states that impose different sociodemographic reporting requirements for different diseases or conditions.

Furthermore, it is important to consider the difficulties of capturing and classifying this type of data in health practice, which may deter health departments from enforcing disease reporting requirements. Studies have documented that health departments prefer to collaborate with, rather than penalize, the providers and facilities they work with in order to promote public health goals.^64^ The fact that the law does not account for the reality that health practitioners face may pose a challenge that public health must solve through technology, policy, and further collaboration. As such, a subsequent study might also include a qualitative component, relying on interviews or surveys of healthcare providers, laboratories, and others tasked with submitting disease reporting information for their perspectives on barriers to reporting sociodemographic data to public health departments. Some identified barriers have included limited or flawed technology, lack of time, or patients opting out of disclosing this information—challenges that CDC’s Data Modernization Initiative seeks to mitigate.^65^ Future research might also include an examination of reporting requirements in light of the specific mechanisms used by reporters to submit this information to public health departments.

## VI. Conclusion

Disease reporting laws serve a vital role in facilitating the creation of comprehensive, illustrative datasets that can be used to monitor, respond to, and prevent future disease and injury. State disease reporting systems contribute to the utility and reliability of NNDSS and similar surveillance systems, which can be employed to better understand, and ultimately correct, disproportionate burdens of disease and injury across populations. A robust collection of sociodemographic information can inform public health professionals and policymakers about how best to target interventions and resources, as well as monitor progress and efficacy of these interventions over time. Sociodemographic information in disease data can identify those populations that face substantial morbidity and mortality risk and that may encounter the most challenging structural and institutional barriers to avoiding or overcoming that risk.

As the world faces new public health threats and emerging infectious diseases with the capacity to spread quickly, the need for early warning systems and rapid public health response becomes even more imperative.^66^ Disease surveillance systems equipped with sociodemographic data can help address the factors that lead to disproportionate health outcomes. To successfully address these inequities, public health must develop tools and an evidence base that accurately depict the myriad ways that sociodemographic factors affect one’s health.

## Footnotes

1. *2020 National Notifiable Conditions (Historical)*, Ctrs. for Disease Control & Prevention, https://ndc.services.cdc.gov/ (choose “2020” from “Year” dropdown menu under “Notifiable Condition Lists”; then click “Get Notifiable List by Year” button) (last visited May 25, 2021). The fifty-seven jurisdictions include the fifty states, Washington D.C., New York City, American Samoa, Commonwealth of Northern Mariana Islands, Guam, Puerto Rico, and U.S. Virgin Islands. *See* Deborah Adams, et al., *Summary of Notifiable Infectious Diseases and Conditions – United States, 2013*, 62 Morbidity & Mortality Wkly. Rep. 1, 7 (Oct. 23, 2015).

2. *National Notifiable Diseases Surveillance System Overview*, Ctrs. for Disease Control & Prevention, https://www.cdc.gov/nndss/docs/NNDSS-Overview-Fact-Sheet-508.pdf (last updated Apr. 19, 2021). *See also How We Conduct Case Surveillance*, Ctrs. for Disease Control & Prevention, https://www.cdc.gov/nndss/about/conduct.html (last updated Apr. 16, 2021) [hereinafter *CDC Case Surveillance*] (“Case notification, in which state and local health departments send deidentified data about confirmed cases of certain diseases and conditions that are tracked nationally to CDC. Case notification is voluntary.”)

3. *See National Notifiable Diseases Surveillance System Fact Sheet*, Ctrs. for Disease Control & Prevention 2 (2018), https://wwwn.cdc.gov/nndss/document/NNDSS-Fact-Sheet-508.pdf.

4. In this article, “reportable diseases” will be used to refer to the list of identified diseases and conditions that are required by state law to be reported to the state or local health departments, while “notifiable diseases” refers to the list of diseases and conditions that jurisdictions voluntarily report to CDC’s NNDSS for disease surveillance.

5. *CDC Case Surveillance*, *supra* note 2.

6. Richard N. Danila et al., *Legal Authority for Infectious Disease Reporting in the United States: Case Study of the 2009 H1N1 Influenza Pandemic*, 105 Am. J. Pub. Health 13, 13 (2015).

7. *CDC Case Surveillance*, *supra* note 2 (stating that hospitals, providers, and laboratories are required to report cases of certain diseases to health departments according to state disease reporting laws, but local and state health departments can voluntarily send deidentified case information to the CDC).

8. Perry F. Smith et al., *“Blueprint Version 2.0”: Updating Public Health Surveillance for the 21st Century*, 19 J. Pub. Health Mgmt. & Prac. 231, 237 (2013).

9. Stephen B. Thacker & Guthrie S. Birkhead, *Surveillance*, *in* Field Epidemiology 39, 41 (Michael B. Gregg ed., 2008).

10. *See, e.g*., Donna Behler MacArthur, *Emerging Infectious Diseases*, 54 Nursing Clinics N. Am. 297, 298 (2019) (discussing generally factors related to the spread of emerging infectious diseases and the difficulties of managing their spread); Vikram P. Krishnasamy et al., *Update: Characteristics of a Nationwide Outbreak of E-Cigarette, or Vaping, Product Use-Associated Lung Injury–United States, August 2019–January 2020*, 69 Morbidity & Mortality Wkly. Rep. 90, 90 (2020) (discussing the mapping of an EVALI outbreak, a lung disease associated with the use of vaping products); Caitlin M. Rivers & Samuel V. Scarpino, *Modelling the Trajectory of Disease Outbreaks Works*, 559 Nature 477, 477 (2018) (noting the importance of modeling and surveillance for addressing pandemics).

11. *See* MacArthur, *supra* note 10; *see also CDC Case Surveillance*, *supra* note 2.

12. *See* Encyclopedia of Research Design 346 (Neil J. Salkind ed., 2010); *see, e.g*., Jonathan W. Dyal, *COVID-19 Among Workers in Meat and Poultry Processing Facilities – 19 States, April 2020*, 69 Morbidity & Mortality Wkly. Rpt. 557, 559 tbl.2 (2020); Jazmyn T. Moore et al., *Disparities in Incidence of COVID-19 Among Underrepresented Racial/Ethnic Groups in Counties Identified as Hotspots During June 5–18, 2020 — 22 States, February–June 2020*, 69 Morbidity & Mortality Wkly. Rpt. 1122, 1123 (2020); Hazel Williams-Roberts et al., *Facilitators and Barriers of Sociodemographic Data Collection in Canadian Health Care Settings: A Multisite Case Study Evaluation*, 17 Int’l J. Equity Health 186, 187 (2018).

13. While the terms may be used interchangeably, some researchers distinguish between them to highlight broader contextual factors and corresponding trends, which is the approach being taken in this Article. *See, e.g.*, Minnesota Health Equity Data Analysis, *HEDA Population Step: Demographic and Socio-Demographic Data for Minnesota Counties*, Minn. Dep’t of Health, https://www.health.state.mn.us/data/mchs/genstats/heda/demographics.html (last visited Apr. 20, 2021) (characterizing “demographic” data as referring to “population by race and age” and “sociodemographic” data as referring to “poverty, graduation rate, and owner-occupied housing”); *see also* Williams-Roberts et al., *supra* note 12.

14. *See, e.g.*, *Health Equity Considerations and Racial and Ethnic Minority Groups*, Ctrs. for Disease Control & Prevention, https://www.cdc.gov/coronavirus/2019-ncov/community/health-equity/race-ethnicity.html (last updated Apr. 19, 2021) (identifying social determinants of health as “the conditions in the places where people live, learn, work, play, and worship that affect a wide range of health risks and outcomes,” and noting that discrimination in “systems” and “social” inequities placed many members of racial and ethnic minority populations at greater risk for contracting COVID-19); *Populations and Vulnerabilities*, Ctrs. for Disease Control & Prevention, https://ephtracking.cdc.gov/showPcMain.action (last updated Oct. 21, 2020) (“Understanding population characteristics is essential for public health practices such as program planning, epidemiologic studies, and public health emergency preparedness. Knowing a population’s characteristics, including their vulnerabilities and resources, can help public health professionals determine possible effects of health problems or environmental conditions on disease trends.”); Megan Daugherty Douglas et al., *Missed Policy Opportunities to Advance Health Equity by Recording Demographic Data in Electronic Health Records*, 105 Am. J. Pub. Health S380, S380 (2015) (discussing the importance of collecting not only demographic data but also granular data in electronic health records to address health disparities); Heather Allen & Rebecca Katz, *Demography and Public Health Emergency Preparedness: Making the Connection*, 29 Popular Rsch. Pol’y Rev. 527, 530 (2010).

15. *See, e.g.*, *Health Equity Considerations and Racial and Ethnic Minority Groups*, *supra* note 14; *Populations and Vulnerabilities*, *supra* note 14; Douglas et al., *supra* note 14; Allen & Katz, *supra* note 14.

16. *Health Equity Considerations and Racial and Ethnic Minority Groups*, *supra* note 14. *See also* Allen & Katz, *supra* note 14.

17. Sonja S. Hutchins et al., *Protecting Vulnerable Populations from Pandemic Influenza in the United States: A Strategic Imperative*, 99 Am. J. Pub. Health S243, S243 (2009). *See also Health Equity Considerations and Racial and Ethnic Minority Groups*, *supra* note 14 (identifying discrimination on the basis of race or ethnicity as a factor that places some people at increased risk for COVID-19); Jennifer S. Middlebrooks & Natalie C. Audage, The Effects of Childhood Stress on Health Across the Lifespan 4 (2008) (describing health outcomes associated with toxic stress, which “results from adverse experiences that may be sustained for a long period of time. This type of stress can … lead to long-term health problems.”).

18. *See* Hutchins et al., *supra* note 17, at S246.

19. This goes beyond merely collecting data on incidences of disease among populations and facilitates the identification of policies, practices, and structural conditions that contribute to disease spread. *Id.* at S243–44. *See also* Sandra Crouse Quinn & Supriya Kumar, *Health Inequalities and Infectious Disease Epidemics: A Challenge for Global Health Security*, 12 Biosecurity & Bioterrorism 263, 268 (2014) (noting that countries can “use the periods between pandemics to examine the mechanisms by which unequal levels of disease and mortality may arise”).

20. Hazel D. Dean, *A Public Health Approach to Achieving Health Equity*, Ass’n of State & Territorial Health Offs.: ASTHO Experts Blog (Apr. 3, 2017, 2:09 PM), https://www.astho.org/StatePublicHealth/A-Public-Health-Approach-to-Achieving-Health-Equity/4–3-17/. *See also* Bruce G. Link & Jo C. Phelan, *Editorial: Understanding Sociodemographic Differences in Health—The Role of Fundamental Social Causes*, 86 Am. J. Pub. Health 471, 471–72 (noting that “societies continue to shape patterns of disease”).

21. James W. Buehler et al., *The Reporting of Race and Ethnicity in the National Notifiable Diseases Surveillance System*, 104 Pub. Health Reps. 457, 458–59 (1989) (noting that states only began reporting in a computer-based network for disease reporting to CDC’s NNDSS in 1985 and identifying variation in the scope of demographic data reported: e.g., for 1987, while ninety-five percent of case reports specified age and ninety-nine percent reported gender, only sixty percent include race-ethnicity). *See also* Nelson Adekoya et al., *Completeness of Reporting of Race and Ethnicity Data in the Nationally Notifiable Diseases Surveillance System, United States, 2006–2010*, 21 J. Pub. Health Health Mgmt. & Prac. E16, E19 (2015); Dean, *supra* note 20 (“[M]any data systems in use have not kept pace with the changing U.S. demographics and need to be updated.”); Alfonso Rodriguez-Lainz et al., *Collection of Data on Race, Ethnicity, Language, and Nativity by US Public Health Surveillance and Monitoring Systems: Gaps and Opportunities*, 133 Pub. Health Reps. 45, 49 (2018).

22. Adekoya et al., *supra* note 21.

23. Chia-ping Su et al., *Case Investigations of Infectious Diseases Occurring in Workplaces, United States, 2006–2015*, 25 Emerging Infectious Diseases 397, 398 (2019). *See also Investigating and Responding to COVID-19 Cases in Non-Healthcare Work Settings: Considerations for State and Local Health Departments*, Ctrs. for Disease Control & Prevention, https://www.cdc.gov/coronavirus/2019-ncov/php/open-america/non-healthcare-work-settings.html (last updated Oct. 21, 2020).

24. Theodore C. Falk, *AIDS Public Health Law*, 9 J. Legal Med. 529, 530 (1988).

25. Danila et al., *supra* note 6.

26. Id.

27. *See* U.S. Dep’t of Health & Hum. Servs., COVID-19 Pandemic Response, Laboratory Data Reporting: Cares Act Section 18115 (Jan. 8, 2021); *COVID-19 Data Reporting for Non-Lab-Based Testing*, U.S. Dep’t of Health & Hum. Servs., https://www.hhs.gov/sites/default/files/non-lab-based-covid19-test-reporting.pdf (last visited June 9, 2021).

28. *See National Notifiable Diseases Surveillance System (NNDSS)*, Ctrs. for Disease Control & Prevention, https://wwwn.cdc.gov/nndss/ (last updated Mar. 13, 2019). Notably, the passage of the Health Information Technology for Economic and Clinical Health (HITECH) Act expanded the use of electronic health records for collecting and recording health data and concomitant demographic data. Over time, observers have noted that while this kind of “federal legislation can be a powerful stimulus for change, its effectiveness in context depends on its ability to accommodate private health care markets, as well as diversity in state and local policies.” Marsha Gold & Catherine McLaughlin, *Assessing HITECH Implementation and Lessons: 5 Years Later*, 94 Milbank Q. 654, 679 (2016). Indeed, as variations between state and local laws and policies governing health data persist long after HITECH implementation, the need for systematic analysis and tracking of reporting requirements continues. *See also* Douglas et al., *supra* note 14, at S380–81, S387 (discussing the HITECH Act’s imposition of baseline requirements to collect limited demographic data and emphasizing the need to collect more granular data in electronic health records to address health disparities).

29. A team of five PHLP researchers conducted a legal scan of disease reporting laws, which included statutes and regulations, for the fifty U.S. states. Researchers first isolated the entire disease reporting chapters from each of the fifty states from a legal database, WestlawNext. They conducted background research to identify patient sociodemographic factors for which data might be needed in order to assess disease burden across populations. This information was incorporated into three coding questions to systematically review each state statute and regulation: (1) does the law require demographic information to be reported; (2) if yes, who is required to report demographic information (health care provider/facility; laboratory; other; N/A); and (3) if yes, what demographic information is explicitly required to be reported (Name, Address, Telephone number, Occupation/employer, Race or ethnicity, Social Security Number, Sex, Age, Date of Birth, Date of Death, Country of Origin, Citizenship Status, Gender, Sexual Orientation, Pregnancy status, Insurance status/payor source, Other, N/A). Researchers included what is arguably also characterizable as personally identifiable information, such as Name, Address, and Telephone Number, because some researchers have considered how they may have utility in the collection of sociodemographic data. *See, e.g.*, David L. Word et al., *Demographic Aspects of Surnames from Census 2000*, U.S. Census Bureau, https://www2.census.gov/topics/genealogy/2000surnames /surnames.pdf (last visited June 9, 2021); Konstantinos Tzioumis, *Data Descriptor: Demographic Aspects of First Names*, Sci. Data (Mar. 6, 2018), https://www.nature.com/articles/sdata201825. Researchers utilized the Public Health Law Information Portal (PHLIP) to code for the presence or absence of each legal attribute included in the three coding questions. Quality assurance checks were conducted by attorney researchers to review each coding decision made for each of the fifty jurisdictions (five states were coded by all five researchers, and all remaining states were double-coded by two researchers.). The validated dataset was then used to assess findings related to mandatory reporting of patient sociodemographic information as part of state disease reporting laws. *See, e.g.*, Tara Ramanathan et al., *Legal Epidemiology: The Science of Law*, 45 J.L., Med. & Ethics (Special Supplement to Issue 1) 69, 69 (2017) (discussing the study of legal epidemiology).

30. *E.g.*, Ariz. Admin. Code § R9-6-204 (2020) (located within the Health Services title of the state’s Administrative Code, this provision contains the clinical laboratory director reporting requirements).

31. This coding scheme captured whether a state had a provision requiring demographic data reporting somewhere within the disease reporting statutes or regulations but did not differentiate a state that required demographic data reporting for *all* reportable diseases and conditions from a state that required such reporting only for a subset of reportable diseases or conditions.

32. *See* discussion *infra* Section V.B.

33. These gaps might be even more pronounced in certain communities. For example, researchers have noted the “excessive absence” of public health data among non-Hispanic American Indian and Alaska Native (AI/AN) persons, highlighting a need for additional case investigation and reporting. Sarah M. Hatcher et al., *COVID-19 Among American Indian and Alaska Native Persons - 23 States, January 31-July 3, 2020*, 69 Morbidity & Mortality Wkly. Rep. 1166, 1167 (2020).

34. Danila et al., *supra* note 6, at 15.

35. *E.g.*, Ala. Admin. Code r. 420-4-1-.04(7) (2014). The two states with no explicit sociodemographic reporting requirements in disease reporting statutes or regulations were Georgia and Tennessee. *E.g.*, Tenn. Comp. R. & Regs. 1200-14-01-.02(1) (2019). One additional state, Oklahoma, required the reporting of demographic information, but did not enumerate any specific markers to be reported; researchers characterized this as requiring the reporting of sociodemographic data. Okla. Admin. Code § 310:515-1-2 (2020) (“The diseases listed in this Chapter must be reported, along with patient identifiers, demographics, and contact information to the Department upon discovery[.]”). *See* additional discussion, *infra* page 8. Therefore, researchers determined that forty-eight states required the reporting of *any* demographic data, and forty-seven states expressly required the reporting of at least an individual’s name and address. Also of note, Wyoming required the reporting of “demographic information” as well as age and gender to its “Colorectal Cancer Early Detection Program,” 048.0046.18 Wyo. Code R. § 2 (2007), and it otherwise required physicians to report sociodemographic data for other cases using an “official State Disease Case Report or equivalent format.” WY Rules and Regulations 048.0046.1 Wyo. Code R. § 6(a) (2001). This Case Report, as with Oklahoma, included sociodemographic data. Wyo. Dep’t of Health, Confidential Disease Report (2012).

36. *E.g.*, Ariz. Admin. Code § R9-6-204 (2018) (requiring laboratories to report name, address, telephone, email, date of birth, and gender for cases of reportable diseases generally, but adding the reporting of race and ethnicity for reporting of positive HIV cases); Cal. Code Regs. tit. 17 § 2504 (2021) (requiring out of state laboratories to report cases of tuberculosis pursuant to § 2500(b), which requires the reporting of demographic data pursuant to § 2500(d)); 048.0046.18 Wyo. Code R. § 2 (2007) (requiring health care providers to report demographic information in conjunction with cases of colon cancer). As researchers set out to determine the extent to which mandates to report sociodemographic data exist at all, the coding scheme for the instant scan was not structured to tie each mandate to specific diseases or conditions. Please see the “Limitations” and “Considerations” sections of this Article for further discussion, *infra* pp. 15–16.

37. *E.g.*, Ala. Admin. Code r. 420-4-1-.04(1) (2014) (identifying physicians, nurses, medical examiners, hospital administrators, laboratory directors, among others to report); Cal. Code Regs. tit. 17 § 2500(b) (2021) (imposing a “duty” on “every health care provider”); N.H. Rev. Stat. Ann. § 141-C:7(I) (2003) (requiring “any health care provider, clinical laboratory director, the superintendent or other person in charge of any hospital, or other health care facility,” among others); S.D. Admin. R. 44:20:02:02 (2011); Tex. Health & Safety Code Ann. § 81.042 (2015).

38. *E.g.*, 007.15.2 Ark. Code R. § III(C) (2019).

39. *E.g.*, Conn. Agencies Regs. § 19a-36-A3(a)(3)(A) (1989).

40. *E.g.*, 10-144-258 Me. Code R. § 2 (A)(5) (2020).

41. *E.g.*, Nev. Admin. Code § 441A.815(1) (1992).

42. *E.g.*, Minn. R. 4605.7070 (2005).

43. *Compare* Ala. Admin. Code r. 420-4-1-.04(7) (2014) (listing specific data to be reported), *with* Okla. Admin. Code § 310:515-1-2 (2019) (omitting any specific data elements to be reported).

44. *E.g.*, 10-144-258 Me. Code R. § 2(B) (2020). As discussed *supra* note 35, the laws examined for Georgia, Oklahoma, Tennessee, and Wyoming did not specify name and address.

45. *E.g.*, Ala. Admin. Code r. 420-4-1-.04(7) (2014).

46. The eight states that did not impose an explicit mandate within disease reporting laws to report racial or ethnic information were Hawaii, New York, North Dakota, Oregon, South Carolina, Vermont, Washington, and Wyoming. *E.g.*, N.D. Admin. Code 33-06-02-01(2) (1996). Of note, Hawaii’s Access to Medical Records regulation states that “every person, health care provider, and medical facility shall provide the patient’s … race or ethnicity … when requested” by the health department during an epidemiologic investigation and the department has the authority to require race or ethnicity be reported by health care providers when the department determines that a dangerous disease outbreak requires monitoring to protect public health. Haw. Code R. § 11-156-4.2(b)–(c) (2009). Researchers did not include this instance as within the scope of a mandatory disease reporting requirement for purposes of this report due to this ambiguity in language.

47. *E.g.*, 007.15.2 Ark. Code R. § IV(A)(3) (2019) (requiring the reporting of sex); Ariz. Admin. Code § R9-6-204(B) (2020) (requiring the reporting of gender); 6 Colo. Code Regs. § 1009-1(1) (2019) (requiring the reporting of both sex and gender). Eight states’ laws required only gender and not sex to be reported, while twenty-seven states required reporting of only sex and not gender. *Compare* Ariz. Admin. Code § R9-6-204(B) (2020) (requiring only the reporting of gender, not sex), *with* 007.15.2 Ark. Code R. § IV(A)(3) (2019) (requiring only the reporting of sex, not gender).

48. Cal. Code Regs. tit. 17 § 2505(b) (2021); Cal. Code Regs. tit. 17, § 2500(d)(1) (2021).

49. *E.g.*, Ala. Admin. Code r. 420-4-1-.04(7) (2014). In addition to Georgia, Oklahoma, and Tennessee, South Carolina only explicitly required the reporting of name and address. S.C. Code Ann. § 44-29-10(A) (2002).

50. These were: Alaska (Alaska Admin. Code tit. 7, § 27.005(c) (2018) (“whether that person is pregnant”)); Arizona (Ariz. Admin. Code § R9-6-202(C)(1)(j) (2018) (“[i]f known, whether the individual is pregnant”)); California (Cal. Code Regs. tit. 17, § 2505(c), (d)(1) (2021)); Florida (Fla. Admin. Code r. 64D-3.030(3)(a)(8), 3.031(3)(a)(8) (2006)); Iowa (Iowa Code Ann. § 139A.3(1)(k) (West 2006); Iowa Admin. Code r 641-1.4(135,139A)(2)(k) (2017)); Kansas (Kan. Admin. Regs. § 28-1-2(d)(1)(H) (2017); Kan. Stat. Ann. § 65-6002(b) (2001)); Kentucky (902 Ky. Admin. Regs. 2:020 (2016)); Louisiana (La. Admin. Code tit. 51, §§ 105(A), 107(A) (2019)); Minnesota (Minn. R. 4605.7090(L) (2017)); Nebraska (173 Neb. Admin. Code, § 1-005.01(D)(7) (2017)); Texas (25 Tex. Admin. Code § 97.3(a)(3)(F) (2017) (for Hepatitis A, B, C, and E)); Utah (Utah Admin. Code r. 386-702-7(1)(a)(ix) (2018)); and Virginia (12 Va. Admin. Code § 5-90-90(A)–(C) (2016)). Some state laws required the reporting of pregnancy status only for certain diseases. For example, Minnesota required pregnancy status and expected date of delivery “if the infection can be transmitted during pregnancy or delivery.” Minn. R. 4605.7090(L) (2017).

51. *See, e.g.*, N.H. Code Admin. R. He-P 301.03 (2016).

52. These seventeen states include: Alabama (Ala. Code § 22-11A-14(b) (2018) (applies to sexually transmitted diseases)); Arizona (Ariz. Admin. Code § R9-6-202(C)(1)(l) (2018) (“[i]f known, the individual’s occupation”)); California (Cal. Code Regs. tit. 17, § 2500(d)(1) (2021) (“if known … occupation”)); Connecticut (Conn. Agencies Regs. §§ 19a-36-A4(a)(1), A11(a) (1989) (“occupation”)); Illinois (Ill. Admin. Code tit. 77, § 696.170(a)(2)(C) (2012) (reporting of occupation applies to tuberculosis cases only)); Louisiana (51 La. Admin. Code tit. 51, § 105(A) (2019) (“occupation, employer information” required for reports of “occupational disease/injury”)); Massachusetts (105 Mass. Code Regs. § 300.180(D) (applies to work-related diseases and injuries only and requires reporting of employer name, place where occupational exposure or injury occurred, and individual occupation if known)); Minnesota (Minn. R. 4605.7090(D)(7) (2017) (“place of work”)); Nevada (Nev. Admin. Code §§ 441A.230(2)(d), 441A.243(2)(d), 441A.815(3)(d) (2011, 2011, 1992) (“occupation, employer … if available”)); New Hampshire (N.H. Code Admin. R. Ann. He-P 301.03(b)(1) (2016) (“occupation and place of occupation”)); New Mexico (N.M. Code R. § 7.4.3.13A(2) (2016) (“occupation”)); New York (N.Y. Comp. Codes R. & Regs. tit. 10, § 2.41(a) (1994) (applies to typhoid)); North Dakota (N.D. Admin. Code 33-06-02-01(2) (1996)); Ohio (Ohio Admin. Code 3701-3-02.1(A)(1) (2014)); South Dakota (S.D. Admin. R. 44:20:02:05(2) (2011)); Texas (25 Tex. Admin. Code § 97.3(a)(3)(I) (2017) (for Hansen’s disease)); and Virginia (12 Va. Admin. Code § 5-90-180 (2004)).

53. N.H. Code Admin. R. Ann. He-P 301.03(b)(1) (2016).

54. These were Illinois (Ill. Admin. Code tit 77, § 696.170(a)(2)(C) (2012)); Michigan (Mich. Admin. Code r. 325.173(13)); and Texas (25 Tex. Admin. Code § 97.3(a)(3)(D) (2017)).

55. Cal. Code Regs. tit. 17, § 2500(d)(1) (2021).

56. It suggests not only reliance upon the individual patient’s knowledge and disclosure of pregnancy status, but also reliance upon the reporter to make the inquiry and document such information.

57. Meghan T. Frey et al., *Surveillance for Emerging Threats to Pregnant Women and Infants*, 28 J. Women’s Health 1031, 1031 (2019).

58. *E.g.*, Martha Anker, *Pregnancy and Emerging Diseases*, 13 Emerging Infectious Diseases 518, 518 (2007). *See also, e.g.*, Deshayne B. Fell et al., *Maternal Influenza and Birth Outcomes: Systematic Review of Comparative Studies*, 124 BJOG 48, 56 (2017); Sascha Ellington et al., *Characteristics of Women of Reproductive Age with Laboratory-Confirmed SARS-CoV-2 Infection by Pregnancy Status — United States, January 22–June 7, 2020*, 69 Morbidity & Mortality Wkly. Rep. 769, 772 (2020) (noting that data on pregnancy status were available for 91,412 women among a sample size of 326,335 women of reproductive age who were positive for COVID-19 and discussing CDC’s creation of a pregnancy surveillance system to fill data-reporting gaps).

59. *See, e.g.*, Eugene Freund et al., *Mandatory Reporting of Occupational Diseases by Clinicians*, MMWR (June 22, 1990), https://www.cdc.gov/mmwr/preview/mmwrhtml/000016 66.htm; Nat’l Inst. for Occupational Safety & Health, Niosh 2008–128, NIOSH Alert, Protecting Poultry Workers from Avian Influenza 7 (Bird Flu) (2008); Marissa G. Baker et al., *Estimating the Burden of United States Workers Exposed to Infection or Disease: A Key Factor in Containing Risk of COVID-19 Infection*, PLOS One, Apr. 28, 2020, at 1, 6.

60. *See, e.g.*, Su et al., *supra* note 23 (noting that workplace diseases are identified via workers, employers, workplace health and safety offices, public health departments that receive case reports from clinicians and labs, and the CDC); Kathleen Kreiss & Kristin J. Cummings, *Field Epidemiology Manual*: *Occupational Disease and Injury*, Ctrs. for Disease Control & Prevention, https://www.cdc.gov/eis/field-epi-manual/chapters/Occupational.html (last updated Dec. 13, 2018).

61. Disease and Injury: Infectious Diseases, Nat’l Inst. for Occupational Safety & Health, https://www.cdc.gov/niosh/docs/96-115/diseas.html#Infectious%20Diseases (last updated June 6, 2014).

62. *Notifiable Disease/Condition Report Form*, Ga. Dep’t of Pub. Health, https://dph.georgia.gov/sites/dph.georgia.gov/files/ADES_DPH%20_Reporting_Poster_Form.pdf (last visited June 15, 2021).

63. Mich. Comp. Laws Ann. § 333.5114(1)(c) (West 2019).

64. *See, e.g.*, Patricia Stone et al., *Impact of Laws Aimed at Healthcare-Associated Infection Reduction: A Qualitative Study*, 24 BMJ Quality & Safety 637, 639–40 (2015); *Healthcare-Associate Infections, Improving Your Access to Electronic Health Records During Outbreaks of Healthcare-Associated Infections*, Ass’n of State & Territorial Health Offs., https://www.astho.org/Toolkit/Improving-Access-to-EHRs-During-Outbreaks/ (last visited June 15, 2021).

65. Brian E. Dixon et al., *Completeness and Timeliness of Notifiable Disease Reporting: A Comparison of Laboratory and Provider Reports Submitted to a Large County Health Department*, BMC Med. Informatics & Decision Making, June 23, 2017, at 1, 7 (discussing study of case reports submitted to a large local health department and finding “provider reporting rates, as well as case report completeness and timeliness, have room for improvement,” and discussing various administrative and process barriers to complete and timely case reporting); *Public Health Surveillance and Data: CDC Data Modernization Initiative*, Ctrs. for Disease Control & Prevention, https://www.cdc.gov/surveillance/surveillance-data-strategies/data-IT-transforma tion.html (last updated Dec. 9, 2020).

66. *See* Peter Nsubuga et al., *Public Health Surveillance: A Tool for Targeting and Monitoring Interventions*, *in* Disease Control Priorities in Developing Countries 997, 998–99 (D.T. Jamison et al. eds., 2d ed. 2006), https://openknowledge.worldbank.org/bitstream/handle/10986/7242/414600PAPER0Di101Official0Use0Only1.pdf?sequence=1.

